# Ambitious but unclear: a policy document analysis of digital cross-sectoral collaboration between social security service and healthcare service in Norway

**DOI:** 10.1186/s12913-026-15234-2

**Published:** 2026-07-29

**Authors:** Maryam Haghshenas, Aslak Steinsbekk, Karen Walseth Hara

**Affiliations:** 1 Department of Public Health and Nursing, Faculty of Medicine and Health Sciences, Norwegian University of Science and Technology (NTNU), Trondheim, 7491 Norway; 2 Norwegian Centre for E-health Research, Tromsø, Norway; 3 Department for Pain and Complex Disorders, St. Olavs Hospital, Trondheim University Hospital, Trondheim, Norway; 4 The Norwegian Labour and Welfare Administration of Trøndelag (NAV), Trondheim, Norway

**Keywords:** Cross-sectoral collaboration, Social security, Healthcare, Policy, Digitalization

## Abstract

**Background:**

Digital technologies are recognized as enablers of collaboration across organizational boundaries. However, the fast pace of technological developments introduces new challenges to policymaking. The study aimed to explore how digital cross-sectoral collaboration between social security service and healthcare service is conceptualized and discussed in Norwegian government white papers.

**Method:**

A qualitative study of documents presenting Norwegian policies regarding digital cross-sectoral collaboration between social security service and healthcare service was conducted. Eleven government reports were used in the dataset.

**Results:**

Most of the documents predominantly focused on the general aspects of collaboration between the two sectors, with comparatively less emphasis on digital solutions. There was a focus in the documents on integrating work-oriented goals within healthcare interventions to improve workforce participation. The potential of digital technologies to enhance cross-sectoral collaboration was mentioned, with an emphasis on the need for robust frameworks and regulatory adjustments.

**Conclusion:**

The limited focus on digital cross-sectoral collaboration between the sectors in the studied documents may indicate the need for prioritization of building collaborative culture before adopting digital tools. A more coordinated and adaptive policy approach is needed to align digital innovation with collaborative goals in order to ensure integrated service delivery across sectors.

**Supplementary Information:**

The online version contains supplementary material available at https://doi.org/10.1186/s12913-026-15234-2.

## Introduction

A large number of working-age individuals in most OECD countries receive social security benefits including income compensation due to health-related conditions [1, 2]. This situation poses major socioeconomic challenges, both at the societal level through increased welfare expenditure, as well as at the individual level by leading to long-term dependency and reduced labor market participation [3, 4]. In addition, literature has shown that work, when aligned with individuals’ abilities and limitations, can contribute to improved well-being and a healthier life [5]. Therefore, there has been growing focus on reducing the number of social security benefit recipients and increasing the number of individuals in the workforce [2, 6, 7]. Given the increasing medicalization within social security services and their growing reliance on health information from the healthcare sector, achieving the goal of increased work participation among people with health-related conditions requires effective cross-sectoral collaboration between social security and healthcare services [2, 8, 9].

As digital technologies continue to evolve rapidly, they have been increasingly recognized as key enablers of collaboration across organizational boundaries [10, 11]. By supporting more efficient information exchange, digital solutions play a crucial role in coordinating services between sectors. In particular, tools such as digital platforms and electronic communication systems have been shown to improve collaboration between healthcare providers and social security services, contributing to more seamless case handling and decision-making processes [12]. However, the fast pace of technological developments introduces new challenges to policymaking [13].

Research has shown that changes in policy can have a profound impact on public health and the welfare of individuals in the society [14]. An example from Norway on policy change regarding digital cross-sectoral collaboration between social security service and healthcare service, is the Individual Placement and Support (IPS), a model of supported employment [15]. Previously, social security employees involved in IPS programs had access to patients’ electronic health records, enabling more seamless coordination between the sectors. However, recent changes in policy implementation have restricted access, potentially altering how these sectors collaborate, which could affect the health and well-being of the population [16].

Despite the significance of the policy discussions, no studies have been identified to examine the policies in relation to digital cross-sectoral collaboration between social security service and healthcare service [17–21].

The study therefore aimed to explore how digital cross-sectoral collaboration between social security service and healthcare service is conceptualized and discussed in Norwegian government white papers.

## Methods

A qualitative study of documents presenting Norwegian policies regarding digital cross-sectoral collaboration between social security service and healthcare service was conducted [22]. The document analysis process was done following the READ approach [23], which is done in the following 4 steps: [1] Ready the material [2], Extract data [3], Analyze data, and [4] Distil the findings. The Standards for Reporting Qualitative Research (SRQR) guideline was consulted in reporting of the study [24].

### Context of the study

#### Social security service

The Norwegian Labour and Welfare Administration (NAV), hereby referred to as social security service, is governed by the Ministry of Labour and Inclusion (Arbeids- og inkluderingsdepartementet in Norwegian). NAV is a collaborative entity between the state and municipalities, responsible for providing social services. NAV’s organizational structure operates through central, regional, and local levels. At the central level, the organization manages policies, fundings, and ensures coordination between NAV units. At the regional and local levels, NAV operates through county offices that implement national policies and coordinate with local offices, and municipal offices that provide direct services to residents [25]. The organization contributes to providing financial security and assisting transition into the workforce for those who are unemployed or have reduced working capacity [25]. The main legal framework includes the Labor and Welfare Administration Act [26], the Labor Market Services Act [27], the National Insurance Act [28], and the Social Services in the Labor and Welfare Administration Act [29].

#### Healthcare service

The healthcare service in Norway is governed by the Ministry of Health and Care Services (Helse- og omsorgsdepartementet in Norwegian). Norway offers universal health coverage for all residents, paid by payroll contributions, state funding, and taxes [30]. The healthcare services encompass primary care, including general practitioners, nursing care, and emergency care, and specialist care, including hospitals, psychiatric and rehabilitation services. The services provided by various levels of the healthcare system must comply with the healthcare regulations in Norway, notably the Specialized Health Services Act [31], and the Municipal Health Services Act [32].

#### Collaboration between social security service and healthcare service in Norway

Collaboration between the Social security Service (NAV) and the healthcare service in Norway is essential to ensure comprehensive follow-up and support for individuals who are temporarily or permanently out of work due to health-related challenges. Although both sectors often engage with the same users, their organizational structures, professional responsibilities, and legal mandates are distinct. This division contributes to sector-specific approaches and practices, often resulting in parallel rather than integrated service pathways [33]. These challenges have gained increasing attention in parallel with the digitalization of public services in Norway, where digital solutions are increasingly expected to support coordination and collaboration across sectors.

### Data collection

The data collection process started with identifying relevant documents to answer the research question. The inclusion and exclusion criteria for identifying the relevant documents for the study are presented in Table 1.

**Table 1:** Inclusion and exclusion criteria for identifying relevant documents

	Inclusion criteria	Exclusion criteria
Type of documents	Reports to The Norwegian Parliament (Melding til Stortinget)	Any other type of governmental documents
Time frame	Documents published from 2014 to 2024	Documents published before 2014
Topic of the documents	Documents containing combination of the following topics • Social security service • Healthcare service • Collaboration between social security and healthcare service • Digitalization	All other topics and documents concerning only one of the following topics: • Social security service • Healthcare service • Digitalization

The reason for including white papers of the type “Report to The Norwegian Parliament” was that these reports incorporate political and economic analysis, and outline initiatives under governmental consideration, with a prospect of being discussed in Parliament and eventually leading to future legislation [34]. The reason for the chosen time frame was to limit to more recent reports which would most likely have a bearing on today’s situation while taking into account the fast development of digital technologies. This time frame also covers the period after the implementation of the Coordination Reform (Samhandlingsreformen) in 2012, which introduced important changes in collaboration between sectors in Norway. Starting the main inclusion period from 2014 allowed inclusion of policy documents developed after the implementation phase of the reform. Moreover, additional publications not found by the main inclusion criteria but related to the study topic were included through citation search. The reason for including additional publications was to ensure that relevant information on topics clearly referred to but not directly covered in the reports meeting the main inclusion criteria still were available in the data set. An example of this instance was the Coordination Reform document, which was submitted to Parliament in 2009, as it was referenced frequently in several of the included reports.

To identify reports to The Norwegian Parliament relevant to the research question, the website “www.regjeringen.no” was searched first in August 2024 and most recently in March 2026, as it is the most comprehensive and trustworthy source for governmental records in Norway. The search involved filtering the documents on the website by document type so that only reports to the Norwegian Parliament were included.

The first author went through the title or short summary of all the reports to The Norwegian Parliament within the last 10 years in order to identify potentially relevant documents that contained information relevant for either social security service or healthcare service were selected to be assessed closely. This resulted in 37 potentially relevant documents. The next step involved reviewing the summaries and table of contents of those 37 documents to identify the ones pertinent to both social security service and healthcare services. This resulted in 12 documents, which were then read in full to assess their relevance to the research question. At this stage, nine documents were identified as relevant for inclusion in the study. In addition, two further relevant documents were identified through citation searches, resulting in a final dataset of 11 documents. The included documents are presented in Table 2. Supplementary file 1 provides access to the original documents.

**Table 2:** The included documents

	Publication year	Document title	Type and status of the document	Chapters included	Name used in the article
1	2009	The Coordination Reform- Proper treatment at the right place and right time [35]	Report to The Norwegian Parliament from the Ministry of Health and Care Services- Finished processing	3 + 14	Coordination reform
2	2015	National Health and Hospital Plan (2016–2019) [36]	Report to The Norwegian Parliament from the Ministry of Health and Care Services- Finished processing	9	Hospital plan
3	2016	NAV in a New Era – For work and activity [37]	Report to The Norwegian Parliament from the Ministry of Labor and Social Affairs (currently the Ministry of Labor and Inclusion)- Finished processing	3 + 5	NAV in a new era
4	2019	Public Health Report (2018–2019) – Good lives in a safe society [38]	Report to The Norwegian Parliament from the Ministry of Health and Care Services- Finished processing	4	Public health report 2019
5	2019	National Health and Hospital Plan (2020–2023) [39]	Report to The Norwegian Parliament from the Ministry of Health and Care Services- Finished processing	4 + 5 + 7 + 8	Hospital plan 2019
6	2021	Strategy for The Field of Work and Health [40]	Report from Directorate of Labor and Welfare and Directorate of Health	2	Work and health strategy
7	2021	No One Left Out – A comprehensive policy to include more people in work and social life [41]	Report to The Norwegian Parliament from the Ministry of Labor and Social Affairs (currently the Ministry of Labor and Inclusion)- Finished processing	5	No one left out
8	2023	Public Health Report- National strategy for reducing social health inequalities [42]	Report to The Norwegian Parliament from the Ministry of Health and Care Services- Finished processing	3	Public health report 2023
9	2023	Mental Health Escalation Plan (2023–2033) [43]	Report to The Norwegian Parliament from the Ministry of Health and Care Services- Finished processing	2 + 3	Mental health plan
10	2024	A Reinforced Work Line - more people in work and fewer on social security [44]	Report to The Norwegian Parliament from the Ministry of Labor and Inclusion- Under processing	1 + 4 + 7	Reinforced work
11	2024	National Health and Coordination Plan- Our joint health service (2024–2027) [45]	Report to The Norwegian Parliament from the Ministry of Health and Care Services- Finished processing	2 + 3 + 9	Coordination Plan 2024

To extract data, the final 11 documents were thoroughly reviewed and searched using various keywords to guarantee the inclusion of all pertinent sections related to the study’s topic. The keyword search was conducted with Norwegian terms, given the materials were in Norwegian. The keywords included variations of names that relate to social security service (NAV, arbeids- og velferdsdirektoratet, arbeids- og velferdsetaten), healthcare service (helse og omsorg, spesialisthelsetjenesten, kommunale helsetjenester), digitalization (digitalisering, teknologi, digital transformasjon, digitale verktøy), and collaboration (samarbeid, samhandling).

Relevant chapters identified through keyword searches and manual reading were extracted and compiled into a separate document. These extracted text chapters constituted the dataset for the thematic analysis. Only chapters relevant to the study aim were included in the analysis. During this process, the first author began identifying preliminary themes within the selected documents to ensure that relevant topics were searched for in all documents [23].

### Analysis

The data was analyzed using practical thematic analysis method [46]. This method involves three main steps: reading, coding, and theming. The analysis was conducted iteratively, with the researchers moving back and forth between these steps throughout the analytical process.

In the first step, the authors familiarized themselves with the data. All authors read summaries of four of the included documents that contained comprehensive information related to the study topic (The Coordination Reform (2009), Strategy for The Field of Work and Health (2021), National Health and Coordination Plan (2024), A Reinforced Work Line (2024)). This approach aligns with recommendations to begin the analysis with a smaller subset of data in order to develop an initial understanding of the material and support a stepwise and systematic analytical process [47]. This provided a shared foundation for discussions and initial reflections on the content of the documents. In addition, the first author then conducted a close reading of the dataset to gain a deeper understanding of the material. During this stage, the authors discussed their initial impressions and reflections on the content of the documents, and analytical memos were written and shared among the authors to support the development of preliminary ideas and interpretations. A notable observation at this stage was that the documents mostly addressed the collaboration between the two sectors, whereas digitization was mentioned to a lesser extent.

The second step was coding. In this step, preliminary themes were first developed through discussions among the authors based on the initial reading of the documents. Following this, the first author reread the dataset more thoroughly and conducted coding by identifying and marking text segments related to the research question and sorting them into preliminary themes. The preliminary themes included regulations, information sharing, work-oriented measures, models, and measures towards digitalization. The coding and the content of the preliminary themes were discussed among the authors and adjusted where necessary. Memos documenting analytical insights were created throughout the review of the documents, as well as in discussions between the authors.

In the third step, the preliminary themes were reviewed and refined through several rounds of discussions among the authors. Some themes were merged, adjusted, or clarified to better reflect the content of the documents and the aim of the study. The authors have different professional backgrounds within health sciences, digital health research, and social security services, which provided complementary perspectives during the interpretation of the material. This contributed to minimizing individual interpretation bias. In addition, preliminary results were discussed with individuals familiar with policy processes, which helped contextualize the findings and encouraged reflection on alternative interpretations.

The final two themes were developed through this iterative process and formed the final content of the result section. The process was deemed complete when the authors agreed that the reviewed documents sufficiently addressed the research question [23]. Quotations illustrating the data were selected and translated into written English by the first author. The translations were revised by the other authors before being used in the final text.

The analysis focused on identifying recurring themes and policy priorities related to digital cross-sectoral collaboration across the included documents. The themes presented below reflect topics that were consistently discussed across several of the policy documents.

## Results

Most of the documents discussed general cross-sectoral collaboration between the sectors, with greater emphasis on this topic in the documents published recently. The prominent topics within the area of digital cross-sectoral collaboration between social security service and healthcare service are organized into the following two main themes in the results: Prerequisite for digital cross-sectoral collaboration and Implementation of digital cross-sectoral collaboration.

Overall, there is a focus in the documents on integrating work-oriented goals within healthcare interventions to improve workforce participation, and the challenges posed by distinct sectoral objectives and regulatory frameworks. Additionally, the potential of digital technologies as tools to enhance service delivery and collaboration between social security service and healthcare is discussed, with an emphasis on the need for updated digital strategies, robust frameworks, and regulatory adjustments to enable effective and secure information sharing across sectors.

### Prerequisite for digital cross-sectoral collaboration

The need for more efficient cross-sectoral collaboration between social security service and healthcare service has been a recurring topic in most of the 11 documents [1–6, 8, 10]. Digital solutions were frequently presented in the documents as a means to address existing challenges in cross-sectoral collaboration and service provision [1, 6, 10]. At the same time, several documents highlighted the necessity of gaining an understanding of the challenges associated with cross-sectoral collaboration, which is mentioned in most documents as a means to facilitate implementation of appropriate measures, including digital solutions [1, 3, 9, 10].

Starting with the oldest document included, the Coordination Reform document from 2009 [1], it has been pointed out that increasing use of social security benefits due to health problems can be a threat to long-term financial capacity of society. This has repeatedly been used as an argument for emphasis on increasing workforce participation [3, 4, 5, 9, 10]. A concrete measure mentioned in some documents [4, 7] was the new work inclusion campaign (*Inkluderingsdugnad* in Norwegian) launched by the government in 2019 in order to include people with reduced workability into job market, with the focus on lowering the threshold for work inclusion for the target group.

Consistent arguments are presented for the need to focus on increasing work inclusion. The divergent objectives of healthcare and social security services have been mentioned in several of the documents as a challenge to achieving this goal [1, 3, 4]. One example from the Coordination Reform document 2009 [1], is that social security service has the goal to assist people with work and benefits, whereas the healthcare service is focused on diagnostics, treatment, and coping strategies. The issue continued to be in focus later on, where the NAV in a New Era document 2016 [3] pointed out challenges related to unclear responsibilities and lack of coordination and cooperation between the social security service and the health sector.

Earlier and closer work-oriented follow-up was a key justification for merging the health-related benefits of rehabilitation benefits, rehabilitation benefits and temporary disability benefits into work-related benefits. This was with the aim of implementing necessary work-oriented measures as early as possible in the course of the illness. At the same time, it was intended to improve the possibilities of combining medical treatment and work-oriented measures (Chap. 3.2.2 in the NAV in a new era)

To address this issue, the Reinforced Work document 2024 [10] suggested prioritization of including work-oriented measures in the treatment process. The stated aim was aligning health treatment goals with employment outcomes and ensuring that patients not only recover from their health issues but would also be equipped to return to work. It was further argued in the same document that doing so will increase the possibility of receiving the right service at the right time while simultaneously following up from both sectors [10]. Similar topics were highlighted as a required focus area in the other documents throughout the years [3, 6].

The Government will strengthen cooperation between the areas of work and health. This means facilitating the greater inclusion of work participation and job mastery as treatment and/or rehabilitation goals in ordinary health and care services and in various patient processes. Health personnel should systematically inquire about and follow up on connections to working life in consultations and in other contact with people of working age (Chap. 4.2.3 in the Reinforced work)

In line with the growing emphasis on work participation, the Reinforced Work document 2024 [10] underscored the necessity of a change in perspective regarding health issues from both sectors. This implies that the collaboration between the social security service and the healthcare service should be instrumental in the identification of additional opportunities for individuals with a variety of diagnoses.

More people with health challenges should be able to participate in working life with the conditions they have. This means, among other things, changing the focus from limitations to opportunities for work and activity – and ensuring the provision of the necessary help to achieve this (Chap. 1.2 in the Reinforced work)

Despite recommendations to prioritize work-oriented measures in the treatment process, the Reinforced Work document 2024 [10] highlighted that such measures are not a stated objective within the healthcare system. The same document referred to the Specialized Health Services Act and Municipal Health Services Act, stating that there are no legal requirements in the existing regulations for healthcare providers to have a focus on work-related measures. These regulatory challenges have also been highlighted in the other documents [1, 6].

Another important topic discussed in the documents was fragmented services. The issue has been pointed out from the Coordination Reform document 2009 [1] and onward as a vital challenge in relation to collaboration, where more combined services were suggested as a focus area. Although the emphasis in the Coordination Reform document 2009 [1] was mostly on services between different levels of the healthcare system, much of what is written is relevant for a barrier to cross-sectoral collaboration between social security service and healthcare service even if not stated explicitly. To enhance the provision of combined social security and healthcare services, the Reinforced Work document 2024 [10] proposed increasing competency development opportunities for service providers in both sectors concerning the scope of collaboration and the prioritization of simultaneous healthcare and work-oriented interventions.

The topic was further discussed in the NAV in a New Era document 2016 [3], which emphasized the need for more coordinated services. An important objective outlined in that document was the early identification of the type of support required for individuals who are either outside the workforce or receiving long-term benefits. This was intended to enable timely and appropriate follow-up, and thereby, contribute to a faster transition into employment. The same document highlighted the relevance of this issue in relation to young people, in light of the growing number of youths receiving health-related benefits in Norway.

Incorporating the guarantee for young people under 20 into a common scheme for young people will mean that this group will not necessarily be offered labour market measures but will receive an offer that is in line with the assessment of the individual’s need for assistance. … This effort may be labour market measures, a combination of labour market measures and education, health treatment, or other targeted follow-up with the aim of transitioning to work or education (Chap. 3.2.2 in the NAV in a new era)

The topic was picked up in more recent documents, including the Reinforced Work document 2024 [10], where the new Youth Guarantee program (*Ungdomsgaranti* in Norwegian) was mentioned, aiming to ensure that unemployed youth receive the requisite support from different involved sectors, emphasizing enhanced coordination between social security and healthcare services [8, 10].

In addition, the Reinforced Work document 2024 [10] suggested developing models in order to provide a structure for and strengthen such collaboration at local, regional, and national levels, with the aim of bridging both disciplines and facilitating the implementation of effective measures. Similarly, this was evident in an earlier document, the Work and Health Strategy 2021 [6], where establishing national guidelines for the existing measures was proposed to improve such collaboration, including the Health in Workplace (HelseiArbeid) and the Individual Placement and Support (IPS) program. No specific existing guidelines were mentioned in the documents.

### Implementation of digital cross-sectoral collaboration

It was observed in several of the documents that over the years, digital solutions have consistently been recognized as both a solution to existing challenges and as an opportunity to ensure improvement in providing services for society [1, 6, 10]. The Coordination Reform document 2009 [1] proposed the use of digital technologies as a general solution to contemporary challenges and highlighted a need for enhanced digital communication among service providers in the future, but did not specify how the implementation should take place. However, this was not mirrored in the other documents included. More recent documents including the Reinforced Work document 2024 [10] and the most recent Coordination Plan document 2024 [11], showed greater emphasis on measures and focus areas to actualize the benefits of the technological solutions.

In developing the ICT policy, it is an objective that electronic communication should be the normal way of communicating. This applies both to the relationship between service provider/therapist and patient/users, and to the relationship between the various service providers/therapists. All documentation and information exchange should take place electronically. (Chap. 3.4.1 in the Coordination reform)

The expected benefits from digital technologies mentioned in the documents primarily pertained to facilitation of collaboration among various stakeholders, improved efficiency of resource use, and enhancement of information exchange among different actors [1, 3, 4]. In addition, the Work and Health Strategy 2021 [6] pointed out that increasing digitalization with the use of register data can increase the opportunity to predict the society’s needs and accordingly, contribute to more decision support in providing individualized services. The same topic was brought up in another document as well [10].

Despite the recurring discussion about digital technologies being beneficial for the collaboration, the only concrete example found in the documents was the modernization program in The Labor and Welfare Administration, mentioned in the Reinforced Work document 2024 [10]. Based on the document [10], the aim of the program was to have better self-service solutions for users of the services, employers and collaborative partners. The argument for renewing the Information and Communication Technologies (ICT) within the modernization program was that the existing systems fail to meet current security standards and regulatory requirements, offer limited adaptability, and provide minimal support for user-friendly services [10].

Several of the documents highlighted that standard practice within the sectors can be challenged after implementing digital solutions. The Coordination Reform document 2009 [1] emphasized the need for organizational changes, new routines and ways of collaboration parallel to the introduction of new technologies within the sectors. The topic has still been in focus in the recent documents, as the Reinforced Work document 2024 [10] pointed out how increasing use of digital technologies can challenge the existing frameworks within which the collaboration currently takes place and argued the need for having safe frameworks for using digital technologies and updated national digitalization strategies to contribute to assuring efficient use of digital technologies in the face of current and future challenges of the society. In addition, the most recent Coordination Plan document 2024 [11] argued for shorter transition time from planning to testing and introduction of digital technologies to facilitate adjustments during implementation.

Regulatory challenges in light of utilizing digital solutions have been consistently highlighted as significant issues in most of the documents [1, 3, 6, 10, 11]. More specifically, inflexibility of regulations has been a contentious topic from the Coordination Reform document 2009 [1] to the latest documents in 2024, where regulations were described as “Not well-adapted for digitalization” in the most recent Coordination Plan document 2024 [11]. In addition, developments regarding evaluation of the legal basis and ethical issues in processing sensitive data between the sectors were suggested to be prioritized in the same document [11].

The regulations have lagged somewhat behind the development of healthcare, organizational and technological developments. Today’s legislation was formulated at a time when healthcare was far less likely to require treatment chains across legal entities. Today’s technology enables the sharing of information across businesses. (Chap. 14.5 in the Coordination reform)

The most recent Coordination Plan document 2024 [11] highlighted recent developments, particularly the requirement for healthcare providers to share health information both within and between organizations, provided certain conditions are met [11]. However, the document also further discussed challenges related to how these regulatory requirements are interpreted and applied in practice. To address this, the need for clearer guidance on interpretation of regulations was highlighted [11].

This leads to regulations being applied differently between organizations and to the fact that it takes a long time to implement solutions. Experience shows that it can take time for the actors to reach an agreement on when the conditions in the regulations have been met. (Chap. 9.5.2 in the Coordination Plan 2024)

## Discussion

It was found that Norwegian policy documents on digital cross-sectoral collaboration between social security services and healthcare services have predominantly focused on the general aspects of collaboration between the two sectors, with comparatively less emphasis on digital solutions. A central policy focus to ensure implementation of appropriate measures has been the integration of work-oriented goals within healthcare interventions to enhance workforce participation. However, this integration is challenged by differing sectoral objectives and rigid regulatory frameworks, which hinder seamless collaboration.

In terms of digital solution implementation, the policy documents underscored the necessity of organizational changes and new routines to support effective digital collaboration between the sectors. Additionally, they mentioned the importance of minimizing the transition time between planning and testing digital technologies. A persistent challenge highlighted throughout the documents was the rigidity of regulatory frameworks and the complexities involved in their interpretation, which continue to act as major barriers to digital cross-sectoral collaboration between social security and healthcare services.

### Digitalization as the solution?

One of the main findings of the study was that digitalization and digital solutions were presented as important but discussed superficially in all the documents over time. Even though the topic was introduced more than ten years ago, discussions have largely remained at a general level. Given Norway’s ambition to become the most digitalized country by 2030 [48], digitalization appears to be an inevitable development in this context. Nevertheless, as the documents primarily emphasized various aspects of cross-sectoral collaboration between social security service and healthcare services, an important question arises: should digitalization be utilized to enhance an already existing collaboration between these sectors, or should it be used to establish a fundamentally new approach to collaboration?

One possible explanation for the limited discussions on digital cross-sectoral collaboration in the documents analyzed could be that the primary priority appears to be the establishment of a collaborative culture between the sectors. Addressing existing structural and cultural barriers may be viewed as a necessary first step before digital solutions can be effectively implemented. Previous research in the healthcare sector has shown that cultural readiness is essential for organizations seeking to realize the full benefits of digital initiatives and to ensure that technological developments contribute to improved service quality [49, 50]. Nonetheless, some documents did acknowledge the potential of digital solutions and suggested that they could play a supportive role in facilitating future collaboration [35, 40, 44].

Another reason for lack of discussions on digital cross-sectoral collaboration could be sector-specific digital initiatives that are currently being implemented independently in Norway, such as the new electronic health record system within the healthcare sector [51] and sick leave follow-up measures in social security service [33]. These initiatives have the potential to transform work processes within each sector, illustrating how digitalization can serve not only as a tool but also as an enabler for improved working conditions and service delivery [52]. However, as highlighted in the results, significant structural, cultural and policy-driven measures are required to enhance digital cross-sectoral collaboration between social security service and healthcare service [53, 54]. Without such foundational improvements, the full potential of digitalization may remain unrealized.

### Imbalance motivations for cross-sectoral collaboration

The imbalanced interdependencies between the social security service and healthcare service could be another factor that may explain limited discussions about digital cross-sectoral collaboration between these sectors in the studied documents. The need for collaboration is more necessary for social security service employees than for the healthcare sector, where dependency on social security service is relatively lower. In the Norwegian context, assignments for enhancing collaboration between the sectors have primarily been assigned to and reinforced in the social security service [25]. Thus, the motivation and incentives for digital cross-sectoral collaboration are likely not equally distributed across the sectors.

Moreover, as the findings illustrated, misaligned objectives between social security service and healthcare services can pose a barrier to effective cross-sectoral collaboration. In the Norwegian context, similar to many other countries [55–57], healthcare prioritization is guided by specific criteria that determine access to services. However, employment-related considerations are not explicitly included in these guidelines. Given the growing body of research demonstrating the positive impact of work on health and well-being [5], the absence of employment considerations in official healthcare prioritization criteria and guidelines used for different diagnostic groups presents a significant barrier to embedding work-oriented measures into standard clinical practice. Including the topic in these guidelines could contribute to shifting the focus of collaboration from “disability assessment to work-capacity assessment”, which has been identified as important for promoting labour market participation among individuals with health problems [2]. Although joint initiatives involving both social security and healthcare services are underway to address these challenges in Norway, there remains a need for stronger policy focus to ensure effective implementation.

### Regulation: a continuous challenge

The rigidity and outdated nature of regulatory frameworks in relation to digital cross-sectoral collaboration could be another reason for superficial discussions around the topic of digital cross-sectoral collaboration within the included documents.

A current example of regulatory limitations in digital cross-sectoral collaboration and data sharing between sectors in Norway is the case of the Individual Placement and Support (IPS) program [58]. New guidelines for the IPS services in Norway have pointed out that IPS job specialists should be employed in and financed by the social security service (NAV), and not the healthcare sector, which influenced access to healthcare providers’ electronic journals, as well as how the collaboration is executed [16]. Despite these limitations, there have been discussions about practitioners finding workarounds to access necessary information. This indicates that when regulations fail to keep up with technological advancements, inconsistencies in service provision may emerge, potentially resulting in unequal access to resources and support for those in need [59]. Therefore, updating regulatory policies to reflect technological advancements should be prioritized on the political agenda, with concrete measures for implementation.

However, the agenda-setting process in policymaking is influenced by the actors involved in drafting and discussing these documents [60]. Given the interdisciplinary nature of cross-sectoral collaboration, it is crucial that all relevant organizations and stakeholders actively participate in the development of regulatory frameworks and policy discussions. Lessons from agile transformation approaches suggest that a more iterative, inclusive, and flexible policy development process could lead to more effective and adaptive regulatory solutions [61]. By ensuring broad stakeholder involvement and adopting a more dynamic approach to policy formulation, digital cross-sectoral collaboration could contribute to achieving more coherent service integration.

### Strengths and limitations

The study offers a novel perspective on digital cross-sectoral collaboration by focusing on the policy level, an aspect often overlooked in existing literature. While the selected documents provided a solid foundation for our analysis, including a broader range of government documents from various administrative levels (e.g., regional or municipal) could have provided additional perspectives on the findings. Furthermore, an empirical study of how these policies are implemented in practice, as well as stakeholders’ interpretations and experiences could offer valuable complementary insights.

### Implications and future directions

The results of this study point to several policy implications. Policy initiatives aiming to strengthen collaboration between social security and healthcare services should be supported by clearer guidance, appropriate funding mechanisms, and incentives that facilitate collaboration as part of everyday practice. In addition, the findings suggest that digitalization should not be understood only as a technological aspect, but also as an organizational and regulatory process that requires clear responsibilities, appropriate frameworks for information sharing, and integration into existing collaborative practices.

Future research should further explore barriers and facilitators for digital cross-sectoral collaboration in practice, including perspectives from professionals, policymakers, and service users. Studies across different welfare and healthcare systems may also contribute to a broader understanding of how digital cross-sectoral collaboration can be supported within different organizational and policy contexts. Although this study is situated within the Norwegian context, the findings may offer relevant insights for other countries with similar organizational structures that aim to strengthen cross-sectoral collaboration through digital solutions and broader digitalization initiatives at the policy and system level. The findings particularly emphasize the importance of organizational, structural, and regulatory conditions in enabling digital cross-sectoral collaboration.

## Conclusion

This study shows that while Norwegian government white papers emphasize the importance of cross-sectoral collaboration between social security and healthcare services, discussions around digital solutions remain superficial and underdeveloped. Despite national ambitions for digitalization, significant structural, cultural, and regulatory barriers continue to hinder digital cross-sectoral collaboration. The findings suggest that without addressing foundational challenges, such as misaligned sectoral goals, imbalanced interdependencies, and rigid regulations, the transformative potential of digitalization will likely remain untapped. A more coordinated and adaptive policy approach is needed to align digital innovation with collaborative goals in order to ensure integrated service delivery across sectors.

## Supplementary Information

Below is the link to the electronic supplementary material.

Supplementary Material 1

## Data Availability

All data analyzed during this study are included in this published article [and its supplementary information files].

## Abbreviations

IPS: Individual Placement and Support
NAV: Norwegian Labour and Welfare Administration
OECD: Organization for Economic Co-operation and Development
PRISMA: Preferred Reporting Items for Systematic reviews and Meta-Analysis
READ: Ready the material, Extract data, Analyze data, Distil the findings
