# Supplementary material for: Ambitious but unclear: a policy document analysis of digital cross-sectoral collaboration between social security service and healthcare service in Norway
Source: BMC Health Serv Res. 2026 Jul 29;26:1474. doi: 10.1186/s12913-026-15234-2 (PMC13700242; doi:10.1186/s12913-026-15234-2)
Supplement: Supplementary file 1 [file 12913_2026_15234_MOESM1_ESM.docx]

Policy documents included in the analysis with direct links to the original sources

- [The Coordination Reform- Proper treatment at the right place and right time](https://www.regjeringen.no/contentassets/d4f0e16ad32e4bbd8d8ab5c21445a5dc/no/pdfs/stm200820090047000dddpdfs.pdf)
- [National Health and Hospital Plan (2016-2019)](https://www.regjeringen.no/contentassets/7b6ad7e0ef1a403d97958bcb34478609/no/pdfs/stm201520160011000dddpdfs.pdf)
- [NAV in a New Era – For work and activity](https://www.regjeringen.no/contentassets/8f9e56528fb340eb9f6515cc21ab5119/no/pdfs/stm201520160033000dddpdfs.pdf)
- [Public Health Report (2018- 2019) – Good lives in a safe society](https://www.regjeringen.no/contentassets/84138eb559e94660bb84158f2e62a77d/nn-no/pdfs/stm201820190019000dddpdfs.pdf)
- [National Health and Hospital Plan (2020-2023)](https://www.regjeringen.no/contentassets/95eec808f0434acf942fca449ca35386/no/pdfs/stm201920200007000dddpdfs.pdf)
- [Strategy for The Field of Work and Health](https://www.helsedirektoratet.no/forebygging-diagnose-og-behandling/forebygging-og-levevaner/arbeid-og-helse/Strategi%202021%20%E2%80%93%20Strategi%20for%20fagfeltet%20arbeid%20og%20helse.pdf/_/attachment/inline/5a1a73df-bed0-4d3c-890f-ff15839ed62a:534106be0bae56cd647d35845769395968a9ed59/Strategi%202021%20%E2%80%93%20Strategi%20for%20fagfeltet%20arbeid%20og%20helse.pdf)
- [No One Left Out – A comprehensive policy to include more people in work and social life](https://www.regjeringen.no/contentassets/ce1f2d9ff9a64db2967817b0b337702c/no/pdfs/stm202020210032000dddpdfs.pdf)
- [Public Health Report- National strategy for reducing social health inequalities](https://www.regjeringen.no/contentassets/918eb71926fc44c8802fe3c2e0b9a75a/nn-no/pdfs/stm202220230015000dddpdfs.pdf)
- [Mental Health Escalation Plan (2023-2033)](https://www.regjeringen.no/contentassets/0fb8e2f8f1ff4d40a522e3775a8b22bc/no/pdfs/stm202220230023000dddpdfs.pdf)
- [A Reinforced Work Line - more people in work and fewer on social security](https://www.regjeringen.no/contentassets/6c89bf4309654a0aaa24a5c6b123b7f1/no/pdfs/stm202320240033000dddpdfs.pdf)
- [National Health and Coordination Plan- Our joint health service (2024- 2027)](https://www.regjeringen.no/contentassets/4e5d9e6c63d24cd7bdab5d8c58d8adc4/no/pdfs/stm202320240009000dddpdfs.pdf)
